# Supplementary material for: Identification of photoperiod-induced specific miRNAs in the adrenal glands of Sunite sheep (Ovis aries)
Source: Front Vet Sci. 2022 Jul 22;9:888207. doi: 10.3389/fvets.2022.888207 (PMC9354845; doi:10.3389/fvets.2022.888207)
Supplement: Supplementary file 1 [file Data_Sheet_1.ZIP › Supplementary file/miRDeep2 file.zip!/miRDeep2 file/Know_Structure/LP42A1/oar-mir-136.pdf]

miRBase precursor	: oar-mir-136
Total read count	: 6716
oar-miR-136 read count	: 1927
remaining reads	: 4789

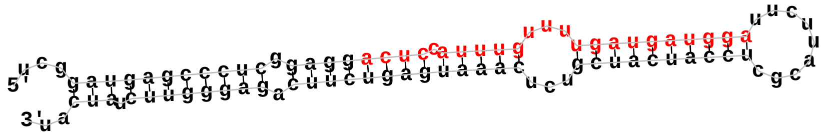

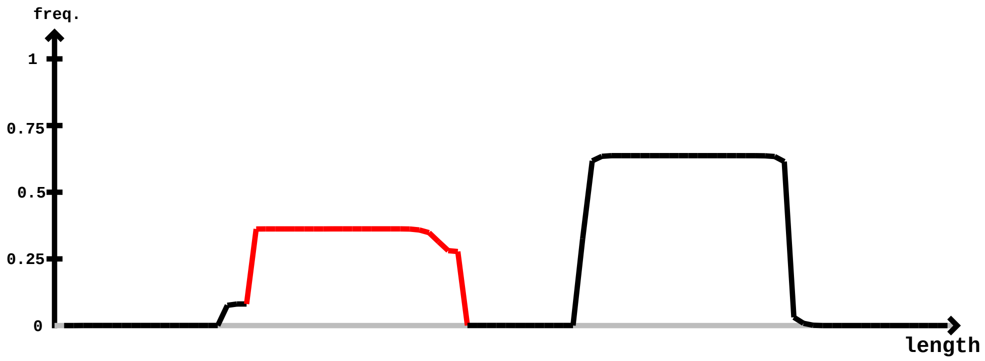

oar-miR-136

```
5'- ucggaugagcccucggaggacuccauuuguuuugaugauggauucuuacgcuccaucaucgucucaaaugagucuucagaggguucuaucau   -3'    exp
    ...(((((((((((.((((((((.(((((...((((((((((.........))))))))))...))))))))))))).)))))))).)))..    reads     mm        sample
    .cggaugagcccucggagg.........................................................................    2         0         seq
    ................aggacuccauuuguuuuga.........................................................    3         0         seq
    ................aggacuccauuuguuuugau........................................................    23        0         seq
    ................aggacuccauuuguuuugaug.......................................................    66        0         seq
    ................aggacuccauuuguuuugauga......................................................    221       0         seq
    ................aggacuccauuuguuuugaugau.....................................................    194       0         seq
    ................aggacuccauuuguuuugaugaug....................................................    1         0         seq
    .................ggaAuccauuuguuuuga.........................................................    1         1         seq
    .................ggacuccauuuguuuugau........................................................    1         0         seq
    .................ggacuccauuuguuuugaug.......................................................    1         0         seq
    .................ggacuccauuuguuuugauga......................................................    7         0         seq
    .................ggacuccauuuguuuugaugau.....................................................    23        0         seq
    ...................acuccauuuguuuugauga......................................................    1         0         seq
    ...................acuccauuuguuuugaugau.....................................................    7         0         seq
    ...................acuccauuuguuuugaugaug....................................................    22        0         seq
    ...................acuccauuuguuuugaugaugg...................................................    1859      0         seq
    ...................acuccauuuguuuugaugaugga..................................................    2         0         seq
    ....................cuccauuuguuuugaugaugA...................................................    1         1         seq
    ....................cuccauuuguuuugaugaugg...................................................    1         0         seq
    ...........................uguuuugaugauggauucu..............................................    1         0         seq
    ..........................................uucuuacgcuccaucaucgucucaaaugagucu.................    2         0         seq
    .....................................................caucaucgucucaaaug......................    1         0         seq
    .....................................................caucaucgucucaaaugag....................    3         0         seq
    .....................................................caucaucgucucaaaugagu...................    15        0         seq
    .....................................................caucaucgucucaaaugaguc..................    90        0         seq
    .....................................................caucaucgucucaaaugagucu.................    2054      0         seq
    .....................................................caucaucgucucaaaugagucuu................    19        0         seq
    ......................................................aucaucgucucaaaugagu...................    2         0         seq
    ......................................................aucaucgucucaaaugaguc..................    35        0         seq
    ......................................................aucaucgucucaaaugagucu.................    1833      0         seq
    ......................................................aucaucgucucaaaugagucuu................    88        0         seq
    ......................................................aucaucgucucaaaugagucuuc...............    6         0         seq
    .......................................................ucaucgucucaaaugagC...................    1         1         seq
    .......................................................ucaucgucucaaaugaguc..................    2         0         seq
    .......................................................ucauUgucucaaaugagucu.................    1         1         seq
```

oar-miR-136

ucggaugagcccucggaggacuccauuuguuuugaugaugagauucuuacgcuccaucaucgucucaaaugagucuucagaggguucuaucau			
....ucaucgucucaaaugagucu.................	32	0	seq
....ucaucgucucaaaugagucuu................	43	0	seq
....ucaucgucucaaaugagucuuc...............	33	0	seq
....ucaucgucucaaaugagucuuca..............	2	0	seq
....caucgucucaaaugagucuu................	1	0	seq
....caucgucucaaaugagucuuc...............	10	0	seq
....caucgucucaaaugagucuuca..............	6	0	seq
